# Playful strategies to foster the well-being of pediatric cancer patients in the Brazilian Unified Health System: a design thinking approach

**DOI:** 10.1186/s12913-021-07018-7

**Published:** 2021-09-18

**Authors:** Leandro Miletto Tonetto, Valentina Marques da Rosa, Priscila Brust-Renck, Megan Denham, Pedro Marques da Rosa, Craig Zimring, Irini Albanti, Leslie Lehmann

**Affiliations:** 1 Graduate Programs in Design and Psychology, Universidade do Vale do Rio dos Sinos, Porto Alegre, Rio Grande do Sul Brazil; 2 Department of Industrial Engineering, Universidade Federal do Rio Grande do Sul, Porto Alegre, Rio Grande do Sul Brazil; 3 Graduate Program in Psychology, Universidade do Vale do Rio dos Sinos, São Leopoldo, Rio Grande do Sul Brazil; 4 Georgia Tech Research Institute, Atlanta, GA USA; 5 Pontifícia Universidade Católica do Rio Grande do Sul, Hospital São Lucas da PUCRS, Porto Alegre, Rio Grande do Sul Brazil; 6 Georgia Institute of Technology, Atlanta, GA USA; 7 Harvard University, Harvard Humanitarian Initiative, Cambridge, MA USA; 8 Dana-Farber Cancer Institute, Boston Children’s Hospital, Boston, MA USA

**Keywords:** Supportive care, Subjective well-being, Design for health, Design for well-being, Design thinking, Service design, User experience, Patient experience, Children, Cancer

## Abstract

**Background:**

Cancer care can negatively impact children’s subjective well-being. In this research, well-being refers to patients’ self-perception and encompasses their hospital and care delivery assessment. Playful strategies can stimulate treatment compliance and have been used to provide psychosocial support and health education; they can involve gamification, virtual reality, robotics, and healthcare environments. This study aims to identify how playfulness, whenever applicable, can be used as a strategy to improve the subjective well-being of pediatric cancer patients in the Brazilian Unified Health System.

**Methods:**

Sixteen volunteers with experience in pediatric oncology participated in the study. They were physicians, psychologists, child life specialists, and design thinking professionals. They engaged in design thinking workshops to propose playful strategies to improve the well-being of pediatric cancer patients in the Brazilian Unified Health System. Data collection consisted of participatory observations. All activities were video recorded and analyzed through Thematic Analysis. The content generated by the volunteers was classified into two categories: impact of cancer care on children’s self-perception and children’s perceptions of the hospital and the care delivery.

**Results:**

Volunteers developed strategies to help children deal with time at the hospital, hospital structure, and care delivery. Such strategies are not limited to using playfulness as a way of “having fun”; they privilege ludic interfaces, such as toys, to support psychosocial care and health education. They aim to address cancer and develop communication across families and staff in a humanized manner, educate families about the disease, and design children-friendly environments. Volunteers also generated strategies to help children cope with perceptions of death, pain, and their bodies. Such strategies aim to support understanding the meaning of life and death, comprehend pain beyond physicality, help re-signify cancer and children’s changing bodies, and give patients active voices during the treatment.

**Conclusions:**

The paper proposes strategies that can improve the well-being of pediatric cancer patients in the Brazilian Unified Health System. Such strategies connect children’s experiences as inpatients and outpatients and may inform the implementation of similar projects in other developing countries.

## Background

Children refer to loneliness and isolation during cancer treatment negatively impacting their childhood [1] and look for positivity while experiencing anger and sadness [2]. Positive emotions are beneficial to pediatric cancer treatment, as they are positively associated with treatment compliance [3]. Against this background, playful interventions involving, for instance, gamification [4–6], virtual reality [7, 8], robotics [9, 10], and healthcare environments [11, 12] have been used to support the treatment of pediatric patients and to promote better user experience [13]. Such interventions can be designed not only for children to “have fun”. They can support psychosocial care and promote health education to help patients deal with cancer and its treatment.

The clinical encounter provides opportunities for pediatric oncology professionals to stage a healing environment [14], in which they (i) listen to the children and answer their questions (emotional support); (ii) explain procedures to them; and (iii) distract them [15]. With such interventions, professionals look for improvements related to well-being issues that are objective (e.g., safety) and subjective (e.g., people’s assessments of their lives) [16].

Subjective well-being refers to patients’ self-perceptions and experiences with the hospital and care delivery [17, 18]; it can be negatively affected by pediatric cancer treatment. Thus, interventions around playfulness can be a valuable contribution to childhood cancer treatment [1–16]. To tackle this issue, this study aims to identify how playfulness can be used as a strategy to improve the subjective well-being of pediatric cancer patients in the Brazilian Unified Health System, known as *Sistema Único de Saúde* (SUS). It focuses on ways to improve patients’ perceptions about themselves, the hospital, and care delivery.

The results may inform service improvement projects that privilege playfulness as means to provide psychosocial support and health education to children and their families. In this study, playfulness does not necessarily entail playing to have fun; it refers to the ludic communicational interfaces (i.e., toys) used with and by the child.

This paper advances the state-of-the-art in supportive care by identifying and discussing how playful strategies can improve the subjective well-being of pediatric cancer patients in Brazil. Even though the current literature indicates how playfulness can be used to foster well-being (e.g., [2, 3, 10, 13]), most studies are focused on the socioeconomic context of developed countries and report isolated, specific interventions (e.g., a game that facilitates the understanding of the disease dynamics). Differently, our study was conducted in a developing country and it focuses on the whole Brazilian healthcare system (SUS). Furthermore, it presents systemic strategies, as opposed to isolated interventions.

### Childhood cancer treatment in the Brazilian unified health system (SUS)

The public healthcare system in Brazil (SUS) seeks to “coordinate and expand coverage to more complex levels of care (e.g., specialist care and hospital care), and implement intersectoral actions for health promotion and disease prevention” ([19], p. 1788). It is widely used by low-income patients. However, when people need complex treatments, such as those required for cancer care, even high-income patients who use private health services commonly switch to SUS [20].

Although cancer treatment does not follow a one-size-fits-all approach in the SUS system, there is usually a pattern in the flow of pediatric cancer treatment. When caregivers observe the first symptoms, they often seek care at Basic Health Units or Emergency Services, which are part of SUS, or through private health insurance [21]. SUS recommends that the first appointment should happen at a primary care facility, polyclinic, or emergency center, where the child will be referred to specialized care [22]. Even though a treatment pattern exists, standards of care are not fully defined. Specific guidelines do not exist, with more investments needed to enhance the care network for childhood cancer [23]. There are also limited resources to provide diagnostic tests and treatment, and little integration of people involved in care and research [24].

The Ministry of Health provides a manual containing the main characteristics of the Basic Health Units, indicating requirements for infrastructure and equipment [25], but these standards are not always met in reality [26]. Most pediatric oncology services in Brazil occur at healthcare facilities that do not have specialized staff and are not accredited by the Joint Commission International (JCI) [24]. The combination of poor infrastructure and incomplete regulations makes SUS a unique system that requires tailor-made strategies to promote the well-being of pediatric cancer patients.

One way to tackle complex issues such as those described above is to gather stakeholders from complementary perspectives to develop service improvement projects [27–32]. This study also employs such participatory research methods involving multiple parties. These will be detailed in the next session.

## Methods

Following a qualitative research approach, this paper draws on the Brazilian Unified Health System as a case study to identify how playfulness can be used in psychosocial support and health education to improve the subjective well-being of pediatric cancer patients. It was approved by the Institutional Review Board of *Hospital de Clínicas de Porto Alegre, Brazil*, where the research took place.

### Sample

Sixteen professionals volunteered to participate in the study. They were designers with expertise in applying creative techniques to systemic challenges (i.e., strategic designers^1^) (*n* = 10), physicians (*n* = 2), psychologists (*n* = 2), and child life specialists (who are also physical education professionals; *n* = 2) with experience with pediatric cancer patients. They had an average of 11 years of experience; 14 held Master’s degrees, two had Bachelor’s degrees, and one had a Ph.D.

Volunteers were accessed via the researchers’ professional networks. They were contacted via email. The message presented the research aims, an invitation to participate in design thinking workshops, and the workshops’ schedule.

### Procedures for data collection

The data collection happened during design thinking workshops. While techniques such as in-depth interviews and focus groups usually involve individuals *reporting* perceptions, attitudes, or behaviors, design workshops are organized around creative activities and thus have a *generative* character [33, 34]. They focus on proposing creative ideas and solutions to a problem, in this case, to foster patient subjective well-being in an interdisciplinary group effort. Design thinking has been widely used in healthcare [34], including recent studies in palliative and supportive care [35].

**Fig. 1:**
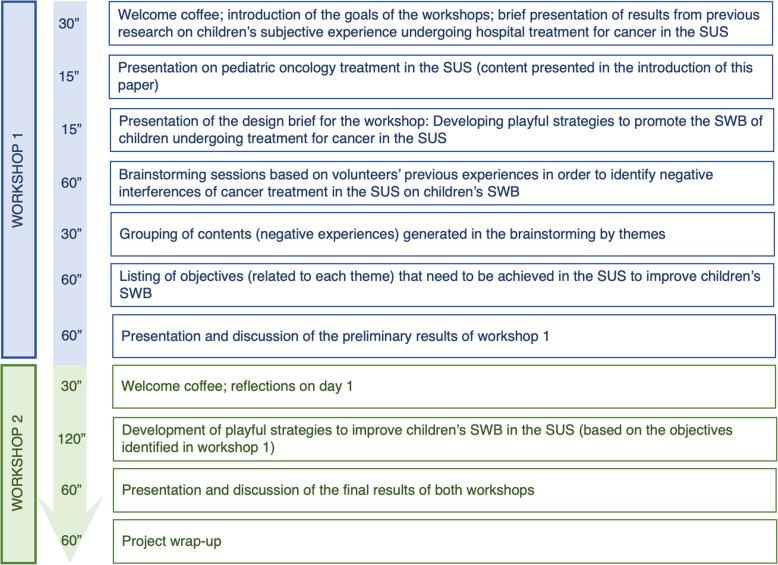
Workshop schedule

The workshops took place at a university building and comprised 2 days of activities, two consecutive Saturdays, that lasted a total of 9 h. Before the activities started, the aims of the research were presented to the volunteers. After having all questions answered, they were invited to read and sign an informed consent form. They were then asked to engage in design thinking activities, following the program described in Fig. 1.

In workshop 1, volunteers were divided into groups, observing a similar distribution of professionals from different backgrounds in each group. Three groups were formed with 5–6 participants. Each group comprised 3–4 design thinking experts and two healthcare professionals; the duos were physician and psychologist, physician and child-life specialist, and psychologist and child-life specialist.

Volunteers brainstormed around a single question: How would you describe your experience related to children’s subjective well-being in the SUS system’s pediatric oncology? The groups were told to avoid reporting commonsense knowledge and interventions already implemented, concentrating their discussion on their experiences in the SUS. They were asked to consider the whole treatment process and any relationships with stakeholders that might impact the children’s well-being. After brainstorming, all contents reporting issues that negatively affect the children’s well-being were organized on a whiteboard, grouped in topics generated by the volunteers themselves. They proposed specific objectives to improve children’s well-being, considering the difficulties described in each topic.

**Fig. 2:**
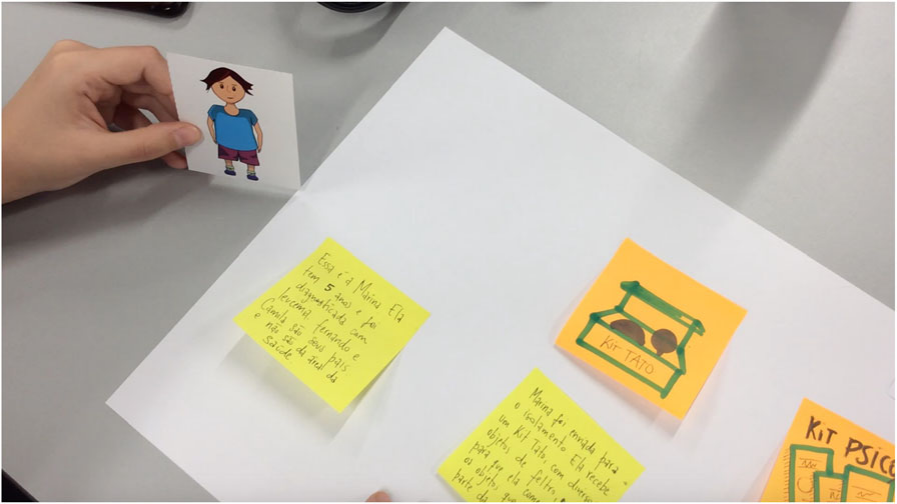
Sample picture of the design process

In workshop 2, volunteers used the available materials (e.g., characters, symbols, and post-its) to develop strategies to achieve the objectives listed in workshop 1. “Stories” applying the strategies were then elicited from volunteers, as exemplified in Fig. 2. They were asked to favor the use of playful strategies as much as possible, following several studies on the topic (e.g., [2, 3, 10, 13]). Other interventions related to psychosocial support and health education that are not grounded solely on playfulness were also considered acceptable. However, they should relate to the playful strategies being designed.

The workshops were video recorded. While volunteers engaged in the design thinking workshops, three researchers with 5 to 20 years of experience in qualitative studies observed their activities and took notes. The notes did not follow a predetermined schedule as the video recordings were used to register all the literal content. The observation modality was participatory, with observers instructed to answer volunteers’ questions about research procedures whenever asked.

### Data analysis

Recordings of the design thinking workshops were transcribed and used in combination with the notes taken by the observers. A Thematic Analysis was carried out manually, enabling the analysis of large amounts of unstructured verbal content [36].

The content produced by the volunteers did not follow a predetermined set of topics. They generated keywords describing issues that negatively affect the children’s subjective well-being and grouped them into themes to summarize the contents. Based on the themes, they established objectives to improve the children’s well-being and strategies to respond to each aim. The researchers later classified all content into two subjective well-being categories from the literature: children’s assessment of the hospital and care delivery and impact of cancer care on children’s self-perception [17, 18]. The recordings’ verbal contents were classified into those categories by two independent researchers who co-authored this paper. When there was disagreement in their categorization, they discussed it and revisited the theory [17, 18] to decide which one was more accurate.

## Results

**Fig. 3:**
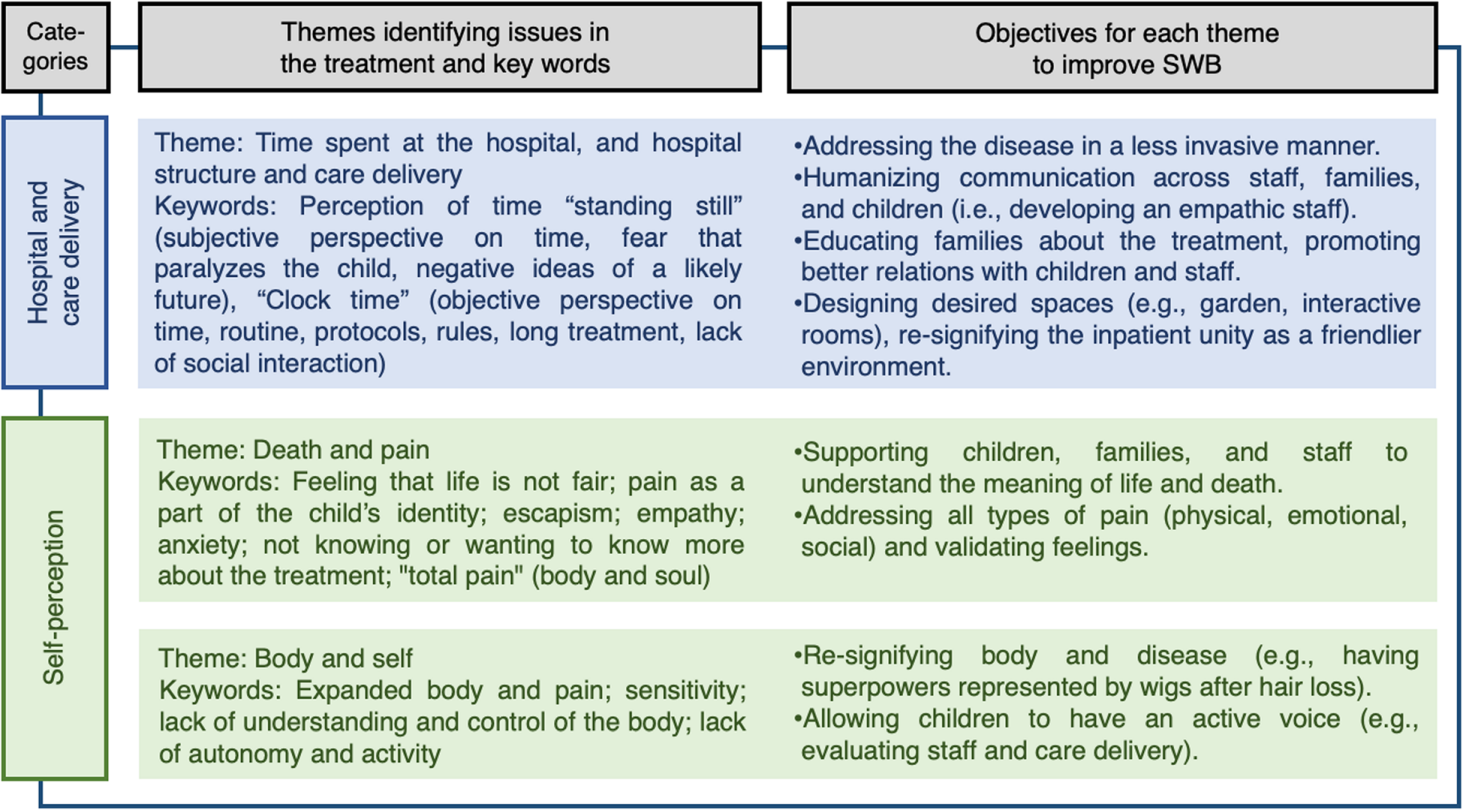
Categories, themes, and objectives to improve patient’s subjective well-being

Figure 3 presents the research results organized by (i) theoretical categories (first column); (ii) themes and keywords generated by the volunteers (second column); and (iii) objectives related to themes for improving subjective well-being, developed during the workshops (third column). Volunteers proposed four strategies to tackle such objectives, labeled “a” to “d”. They are presented within the text, as they do not relate to single themes.

In addition to the three themes – time spent at the hospital (related to the category hospital and care delivery), death and pain, and body and self (both associated with the category self-perception) – communication [1–3] was also deemed by volunteers as cross-cutting and absorbed within the three existing themes. It is briefly described in the following subsection as the topic will appear in the discussion of all the other main topics observed in Fig. 3.

### Cross-cutting theme: communication

The volunteers perceived communication as a fundamental theme and created a persona and fictional story to illustrate it. According to one of the designers, the persona (Marina, a five-year-old girl diagnosed with leukemia) and her parents had “no clue about what would happen [during the treatment], as they did not have previous knowledge about the disease.” Shortly after being admitted to the hospital as an inpatient, she received a kit with objects such as “toy versions of artifacts that were to be used by the healthcare team during her journey at the hospital”, from simple syringes to IV poles. “She attributed new names to medical equipment, e.g., the IV pole that she called ‘doggy’, as she had to take its leash.” She had small objects such as bottoms to communicate feelings and gift people she likes and treat her well.

**Fig. 4:**
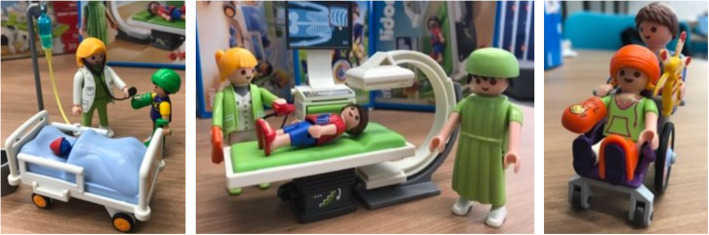
Toy versions of patients, health care team, and medical artifacts

In the story told by the volunteers, the objects were interfaces that would support the healthcare staff in explaining the disease and the treatment, using developmentally appropriate language to communicate with Marina and her family. Examples can be observed in Fig. 4.

Such toys may be used in medical play to support children in normalizing and coping with medical equipment and treatment procedures. By applying a potentially playful communication process, the children’s subjective well-being can be increased as they develop an understanding of the disease, adopt an active voice, and gain a sense of control during the treatment [14, 15].

In this case, ‘playful’ refers to the ludic interfaces used to communicate with children; the activities involving them are not necessarily amusing. Yet, the interaction with such toys may be a moment of play and fun; the children control how they will interact.

As mentioned earlier in the text, communication is connected to and observed in all other themes. The following subsections describe the themes related to the two theoretical categories (i.e., children’s assessment of hospital and care delivery and self-perception).

### Hospital and care delivery

The first theoretical category - “hospital and care delivery” - revealed perceptions of time passing at the hospital as a core theme in the analysis, evidencing the negative feelings children experience going through hospital cancer treatment [1–3]. To create a friendlier environment, healthcare professionals should consider the patients’ needs to “create a similar scenario to what they live at home with friends” (Physician). Patients should experience the hospital as “healing institutions” (Psychologist), providing services focusing on improving their subjective well-being.

To address the aims related to this first category, strategy “a” is to design artifacts to systematically collect and analyze data about subjective well-being (e.g., toy robots that identify facial expressions) (e.g., [13]). The artifacts should perfect themselves based on data collected during the treatment through machine learning tools.

“We should embrace their whole journey as patients and collect data to understand their levels of well-being throughout the entire treatment. Can we use cute robots or some other technological alternative to do that automatically? (…). If we can implement something like that in the SUS system, we could help that particular child and also learn how to respond to situations that are somehow similar [with other children]. We would learn more about how to fight [adverse situations] with the resources we have. (…). We don’t have enough staff to collect that [data] manually. Machines [i.e., machine learning] could support it and accelerate service improvement projects.” (Child life specialist).

This strategy does not imply that healthcare staff should not be trained to recognize and deal with children’s emotions. The artifacts, as mentioned earlier, represent complementary alternatives that help track changes in children’s emotions throughout the day as professionals such as child life specialists are not with them the whole time.

Such artifacts also support healthcare professionals in collecting data as they are a computerized, automatic way of tracking children’s evolution through treatment. They are alternatives that could help provide a more comprehensive understanding of children’s experiences during the treatment; information across children, families, and professionals could be triangled to allow service improvement projects and facilitate communication flow.

Examples of playful interventions related to this strategy are mobile apps with games to allow *face recognition* and detect mood variations and social robots for patients to play with while registering children’s speech through *voice recognition* to identify variations in their emotions (positive and negative words). In this case, a toy robot or similar product would be able to stimulate emotional expression by playing with the child or telling funny stories to evoke joy, thus evidencing its potential to be playful. The child’s responses (e.g., apathy or excitement) and their engagement in play would help understand their emotions.

Manual data collection is also a way to implement such a strategy; however, it is time-consuming. E.g., physical toys (e.g., balls) with *movement trackers* would allow observing vitality (a wellness indicator), and board games would facilitate children symbolizing their reactions to the treatment by showing how characters feel at a hospital.

By addressing the needs of children based on their assessment of the hospital and care delivery, their self-perception, which is the following category, can also be improved.

### Self-perception

The second category – “self-perception” – was divided into two themes: “death and pain” and “body and self”. In the theme “death and pain”, volunteers stated that children should be treated as a whole, with professionals addressing their “total pain” (Physician) [2, 3, 8, 9, 14, 15]. Participants believe there is an unconscious “silence conspiracy” (Psychologist) in the SUS, which prevents death from being discussed; talking about the emotions evoked by finitude is largely avoided: “Support should be offered for children to develop ‘emotional literacy’ that may help them understand, name, and validate feelings.” (Physician). As to the theme “body and self”, volunteers reported the lack of agency some children experience during cancer treatment, with their reduced abilities to control their bodies and limited autonomy [2, 3]. Three strategies (“b”, “c”, and “d”) were developed by the volunteers and addressed the two themes as a whole.

Strategy “b” is to design artifacts to support children, families, and professionals to improve subjective well-being collectively (e.g., using virtual reality to visualize what happens inside the body during the treatment) (e.g., [5, 9]).

“If we can use ludic language, like in virtual reality, to show in a symbolic language what is going on, the treatment will be a lot easier on them. (…). It is not wrong to feel bad. We probably should not say things like: ‘Don’t worry. It will be fine.’ Let them feel what they feel and help them understand why they are feeling a certain way. No one should tell them how it is to be in that situation.” (Designer).

To apply playfulness in this strategy, the volunteers suggested alternatives such as having children visualize “the path that medicine ‘drops’ [chemotherapy drugs] take through their veins like in a roller coaster ride.” (Designer). In this example, virtual reality could be effective, yet it is the most obvious choice; physical artifacts may play similar roles. Analogic examples are (i) educative books using ludic language and characters to represent children during cancer treatment, to be read by parents and patients together; (ii) toys to personalize how IV poles look like aiming to reduce the negativity often associated with the equipment (e.g., character heads to be used on the top of the pole and to be named as a friend or a pet by the children, having alternative faces according to the patient’s mood); (iii) fun-looking, quickly cleanable bedside tables’ surfaces changed according to its different uses (e.g., to minimize the child’s rejection towards food, as the table is frequently associated with medicine and pain, according to the volunteers).

Standardized solutions are not ideal in the SUS environment [21, 22]. With that in view, strategy “c” comprises tailoring projects according to the evaluation of needs and preferences of each child before admission, connecting life in and out of the hospital [29]. Such projects should start after diagnosis and be extended throughout the hospitalization.

“Neither the treatment necessarily finishes when they are dismissed from the hospital, nor their psychological pain. They face many hardships trying to get back to their normal lives while continuing their treatments [such as] going back to school and explaining what is happening to friends. Our [healthcare] system is all dissociated: How come we do not communicate with the educational system, too? They are dismissed from the hospital, and that’s it.” (Child life specialist).

According to the volunteers, technology can help diminish the negative effect of isolation from the child’s social context while respecting the likely need for remoteness in some stages of cancer treatment. E.g., tailoring educational content can be easily achieved with the many technological alternatives, such as personalized image and photo sharing to support children’s individual needs while hospitalized [29].

In addition to personalized educational content that would be shared asynchronously, synchronicity is desirable to keep personal bonds. E.g., classmates taking turns could be “teachers for a day”. Instead of attempting to connect the child online to an in-loco class with dozens of colleagues, when they might not be feeling well, the “teacher” can meet the “student” (patient) to update on the classes at a time of their preference. The encounter may not have a content-driven approach; it may be a source of joy based on play, motivation, and connectedness.

An example of playful intervention focusing on maintaining friendships is to design sterilized yet personalizable puppets that can be shared between patients and their friends out of the hospital. Such toys could have accessories (e.g., a backpack with personal content) and be drawn on and be colored to spend a weekend with the patient’s friend, accompanied by a camera to connect live with friends, make films and take pictures. The puppet could do other playful activities like dancing with friends at a school party (on camera), dramatizing amusing situations when playing at the “remote school”, and dress-up challenges.

Finally, strategy “d” is to have professionals that are not employed by the hospital as service providers since the amount of work and the intense interaction with children may lead to difficulties in addressing them in a humanized manner (e.g., leading to burnout) [21]. “We do not have people to do these things (…) robots, virtual reality. Maybe we will need to rely more on our academic partners. We already have so much going on. It is so difficult to handle all this, so I cannot say that this is a good idea if we have to do it all by ourselves.” (Physician).

According to the volunteers, “it takes time to be at ease” and humanize relationships by being playful (Child life specialist). Therefore, there is no specific playful intervention to be mentioned solely related to this strategy. The volunteers considered it as a requirement to implement all previous strategies.

The results indicate strategies around playfulness that were brought up by the volunteers as having the potential to increase subjective well-being. Such strategies targeted improving children’s self-perception and assessments on the hospital and care delivery by specifically addressing the needs of pediatric cancer patients in the SUS system. In what follows, each strategy is discussed, and suggestions for implementing them in the SUS system are provided.

## Discussion

The use of expensive technologies is typically cost-prohibitive due to SUS’ chronic underfunding. Nevertheless, universities, industry, and government working collaboratively may make it possible to develop such technological projects [37, 38]. Examples of collaboration are medical and industrial design schools working together to provide qualified work, research grants from governmental agencies, and philanthropic actions to fund the work and materials needed.

Strategy “a” was developed to collect and analyze data about subjective well-being systematically. Having access to the evaluation of users’ perceptions of hospitals and care delivery is likely to foster quality improvement projects. Technological playful artifacts that enabled recurrent interactions with the children were mentioned as data collection and analysis tools.

The first step to implement this strategy would be to assess patients’ experience with the infrastructure and equipment in the SUS environment, based on parameters defined by the Brazilian Ministry of Health [25]. The second step would be to design service improvement projects through participatory processes (e.g., [30–32]), bringing together governmental agencies, communities, and the private sector [39]. An example of a current joint initiative is private hospitals offering grants to fund public health research, deducting the research-related expenses from their taxes. Another example entails universities offering training to pediatric cancer patients’ parents in activities that allow them to have a legal source of income while they stay away from their hometowns during their children’s treatment.

Strategy “b” comprises the development of artifacts to improve communication among children, families, and professionals, as well as patients’ understanding of the disease. Health professionals commonly have difficulties finding the time to communicate effectively with patients and patients’ families due to work overload in the SUS. As a result, to mention only one, simple questions from children and families about the treatment are not being addressed.

Knowledge sharing is crucial to increase children’s subjective well-being, especially considering that many Basic Health Units in Brazil do not have specialized staff and facilities [24]. Technology is increasingly playing an essential role in promoting knowledge about cancer. It offers children a ludic way to visualize what cancer is and what to expect during the treatment. Likewise, technologies such as mobile applications may increase communication, but simpler solutions, such as whiteboards available at hospital corridors, may also work in this regard.

Strategy “c” states the need for projects tailored to each individual to promote subjective well-being. In developing such projects, it is important to consider all treatment stages, from diagnosis to cure. Specific interventions (e.g., designing virtual reality to assist in health education during hospitalization) are not comprehensive enough to tackle the complexity of subjective well-being in the SUS system. The lack of healthcare protocols specifically addressing humanization [26, 40] makes children’s experiences even more challenging.

Strategy “d” refers to the aforementioned desired collaboration between hospitals and other parties (i.e., professionals from non-governmental organizations and philanthropic institutions, scholars, and/or graduate students). According to the volunteers, the services described in Fig. 3 should be provided by professionals not employed by the SUS. There are at least two advantages to this service model. First, professionals that do not partake in everyday hospital routine are less likely to suffer from difficulties such as burnout syndrome. Second, SUS’s staff does not include certified technical professionals capable of designing complex technologies; it could only be achievable through interinstitutional collaboration.

**Fig. 5:**
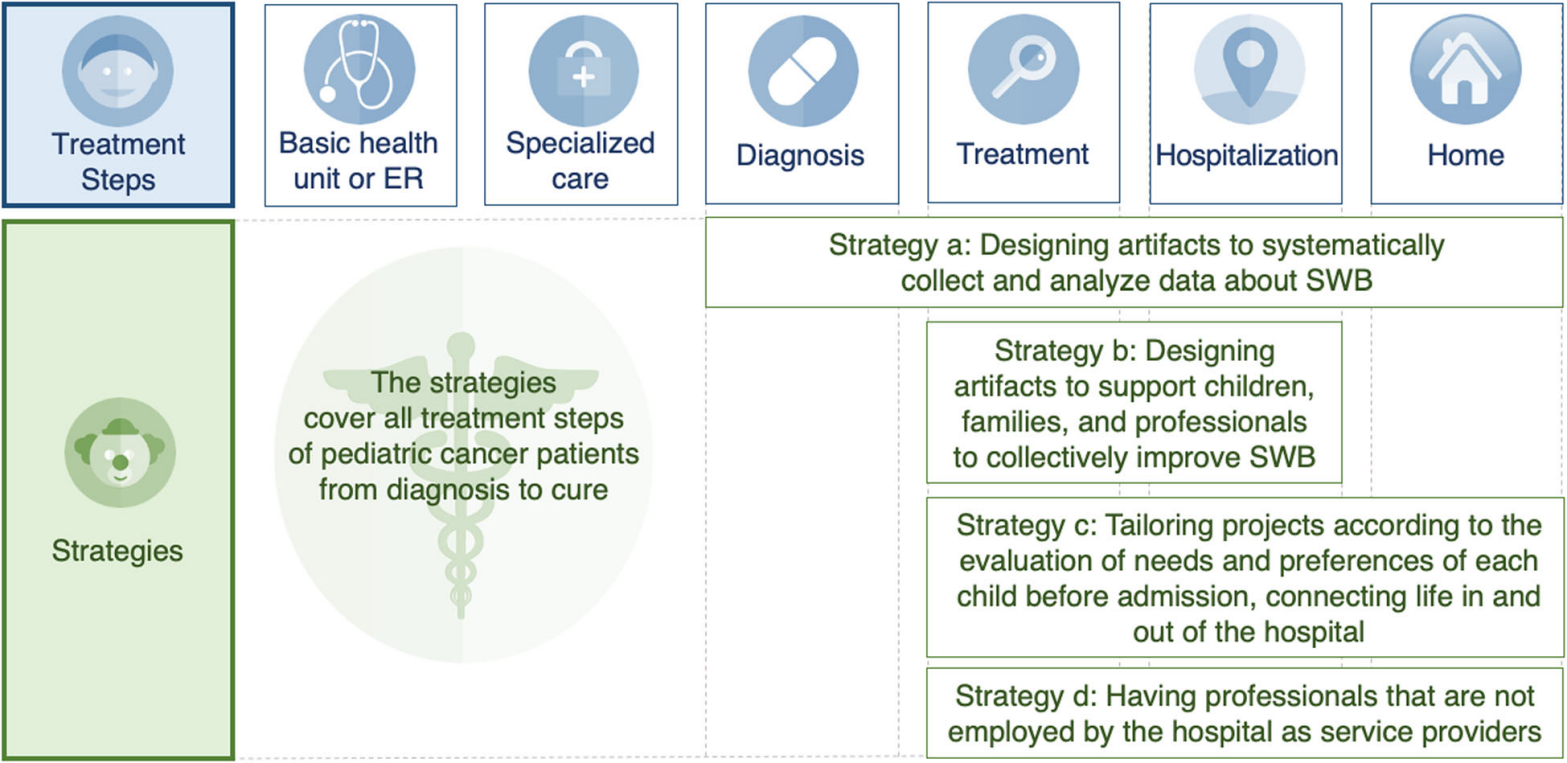
Treatment stages and strategies to improve subjective well-being

Figure 5 summarizes the treatment steps and the proposed strategies to improve subjective well-being.

### Challenges and steps to apply the results in the SUS system

To be applicable in the entire SUS system and not only in isolated hospitals, the well-being improvement project needs to be feasible on a large scale. Since it is an academic initiative, the starting point will be academia, but implementing the strategies presented in Figs. 3 and 5 depends on joint efforts. The following steps will be required to implement the project:

- A leading university will start the project. In this case, it is the institution to which the first author of this paper is affiliated. An initial grant was awarded to the author by the Coordination for the Improvement of Higher Education Personnel (*Coordenação de Aperfeiçoamento de Pessoal de Nível Superior –* CAPES) to conceptualize the approach presented in this paper. Thus, a governmental agency is already supporting this project, which facilitates access to the SUS system.
- The leading university will (i) apply for additional grants to allow for the implementation of the project in other SUS hospitals, in addition to the two that are already being benefited by the project; (ii) manage the project and provide the human resources needed to develop the strategies into tangible solutions (e.g., designing toy-robots), and (iii) implement them at local hospitals.
- The leading university will form a consortium of universities. Each will deal with the implementation of the project in one or more local hospitals. A second University has already partnered with the research team, for that matter.
- The main SUS hospitals providing pediatric cancer care will be contacted and connected to a university. Geographic distance is a significant issue in large countries with remote areas, so proximity will be an eligibility criterion to connect universities to hospitals.

It should be highlighted that measurements of children’s subjective well-being need to be taken at each hospital before and after implementing each strategy. Repeated measures should be used to track the evolution of subjective well-being through time and provide feedback to improvement teams.

### Limitations

The main limitations of this study are fourfold. First, the study only included strategic designers focused on healthcare services, mental health professionals, and child life specialists from Brazil. Future studies are needed to include parents and children to validate the strategies. Second, much of the strategies developed in our study refer to what child life specialists would do in Countries such as the United States. Therefore, exploring strategies around playfulness with these professionals is important in future research. Third, volunteers were experienced professionals from benchmark hospitals in Brazil. Professionals with experience at hospitals with more deficient infrastructures (e.g., those with fewer resources located in rural areas) should be included in future work. Fourth, it should be highlighted that playfulness is one of the many ways to support pediatric cancer patients’ psychosocial care and health education. Our study emphasized play, but further research may expand its scope.

## Conclusions

Even though this research draws on the Brazilian Unified Health System as a case study, the main contribution of this paper is to inform the implementation of similar projects in large-scale healthcare systems of other developing countries, as children’s wellbeing is affected by factors such as ethnicity and language [41]. The coordination of efforts and the lack of human and financial resources that characterizes the SUS make it a rich learning context.

The working model adopted in the earlier version of this project currently being implemented (see discussion section) still concerns mostly discreet solutions (e.g., informative books). Although such solutions work towards subjective well-being, the volunteers indicated that it is mandatory to adopt a systemic approach that takes into account children’s experiences as inpatients and outpatients in the SUS.

## Data Availability

Data used in the study are transcripts of design workshops. They are not available due to a lack of consent to share whole transcripts from participants.

## Footnotes

1 Some design schools in countries such as Italy, Finland, and Brazil offer degrees in Strategic Design. Strategic designers develop innovative product-service systems (e.g., products, associated services, and communication), instead of focusing on creating discrete solutions (e.g., products, buildings, services).
